# Exploring the Contextual Factors That Influence Polio Supplementary Immunisation Activities in the WHO African Region: A Rapid Review

**DOI:** 10.3390/vaccines13080870

**Published:** 2025-08-16

**Authors:** Abdu A. Adamu, Duduzile Ndwandwe, Modjirom Ndoutabe, Usman S. Adamu, Rabiu I. Jalo, Khalid Abubakar, Johnson Muluh Ticha, Samafilan A. Ainan, Messeret Shibeshi, Terna Nomhwange, Jamal A. Ahmed, Charles Shey Wiysonge

**Affiliations:** 1 Polio Eradication Programme, World Health Organization Region Office for Africa, Djoue, Brazzaville BP 06, Congo; ndoutabemo@who.int (M.N.); uadamu@who.int (U.S.A.); tichaj@who.int (J.M.T.); ainans@who.int (S.A.A.); nomhwanget@who.int (T.N.); ahmedjam@who.int (J.A.A.); 2 Vaccine-Preventable Diseases Programme, World Health Organization Regional Office for Africa, Djoue, Brazzaville BP 06, Congo; sheyc@who.int; 3 Cochrane South Africa, South African Medical Research Council, Francie van Zijl Drive, Parrow Valley, Cape Town 7500, South Africa; duduzile.ndwandwe@mrc.ac.za; 4 Department of Community Medicine, Bayero University/Aminu Kano Teaching Hospital, Along Zaria Road, Kano 700101, Nigeria; rabiuibrahimjalo@yahoo.com; 5 Strategic Health Information Cluster, World Health Organization Kogi State Field Office, Lokoja 270001, Nigeria; isahk@who.int; 6 Department of Paediatrics and Child Health, Muhimbili University of Health Allied Sciences, Dar es Salaam 65001, Tanzania; 7 Immunisation, Vaccine Preventable Diseases and Polio Transition, World Health Organization Regional Office for the Eastern Mediterranean, Cairo P.O. Box 7608, Egypt; eshetum@who.int

**Keywords:** poliomyelitis, novel oral polio vaccine type 2(nOPV2), supplementary immunisation activities, contextual factors, implementation determinants, Consolidated Framework for Implementation Research, systems thinking, Africa

## Abstract

**Introduction:** Polio supplementary immunisation activities (SIA) are implemented to rapidly increase vaccination coverage and interrupt the transmission of poliovirus in a specified geographical area. Polio SIA complements routine immunisation and is crucial for the eradication of the disease by increasing population immunity. However, several contextual factors (i.e., implementation determinants) can influence the success or failure of polio SIA implementation; as such, understanding their dynamics can enhance proactive planning for practice improvement. This study aimed to explore and map the contextual factors of polio SIA implementation in the African region using a critical systems thinking approach. **Methods:** A rapid review of published and grey literature was conducted. The search included the Global Polio Eradication Initiative library for programmatic reports and two databases (PubMed and Google Scholar). Data extraction was performed using a structured tool. Thematic analysis was performed to categorise the identified contextual factors according to the domains and constructs of the Consolidated Framework for Implementation Research (CFIR). Then, a causal loop diagram (CLD) was used to map the linkages between the identified factors. **Results:** A total of seventy-eight contextual factors across the five CFIR domains were identified: three for innovation, twenty for outer setting, sixteen for inner setting, twenty-six for individuals, and thirteen for the implementation process. A system map of all the factors using CLD revealed multiple contingent connections, with eleven reinforcing loops and four balancing loops. **Conclusions:** This study identified the multilevel nature of the contextual factors that influence polio SIA, including their dynamics. The integration of CLD and CFIR in this study offers critical insights into the potential feedback loops that exists between the contextual factors which can be used as leverage points for policy and practice improvements, including tailoring strategies to enhance polio campaign implementation effectiveness, especially with the expanded use of the novel Oral Polio Vaccine type 2 (nOPV2) across countries in the region.

## 1. Introduction

The enormous effort and investment in poliomyelitis have undoubtedly yielded significant health gains and accelerated progress towards the global eradication of the disease [1]. The incidence of polio has reduced by more than 99.9% compared to when the World Health Assembly established the Global Polio Eradication Initiative (GPEI) in 1988 [1]. While types 2 and 3 of the wild poliovirus (WPV2 and WPV3) have been certified as eradicated globally, type 1 (WPV1) is currently restricted to two geographies: Pakistan and Afghanistan [2]. At the end of the last decade, the World Health Organization (WHO) African Region received its certification as being free of all indigenous wild polioviruses [2]. However, the emergence and spread of circulating vaccine-derived polioviruses (cVDPVs) across the African region pose an epidemiological threat to achieving the elimination of all poliovirus variants in circulation [3,4].

The spread of polioviruses is a serious global health issue as it is considered a Public Health Emergency of International Concern (PHEIC) under the 2005 International Health Regulations (IHR) [5]. Once detected, a set of actions must be instituted immediately [6]. The GPEI recommends that at least two high-quality supplementary immunisation activities (i.e., vaccination campaigns) be completed within eight weeks of detection using an oral polio vaccine [6]. The purpose of polio supplementary immunisation activities (SIA) is to rapidly improve polio vaccination coverage in the geographical area where the variant has been detected to stop the outbreak (i.e., break the transmission of the disease) [6]. Several countries in the African region with circulating vaccine-derived polio virus type 2 (cVDPV2) are conducting SIA with the novel oral polio vaccine type 2 (nOPV2), which contains a genetically stable viral strain with less chance of reversion [7,8,9].

To be effective, each polio SIA needs to be of high quality with vaccination coverage greater than 95%, validated by an accepted Lot Quality Assurance Survey (LQAS) which must be attained at the sub-national level [6,10]. LQAS is a rapid post-campaign survey to assess the quality of SIA by determining whether the minimum vaccination coverage is reached in a defined geographical area, so as to inform mop-up campaigns [11]. However, it has been observed that in a few sub-national areas within a few countries in the African region, this quality benchmark for polio SIA is sometimes not met [12,13]. An example of an SIA assessment conducted in Nigeria revealed widespread variation in quality between local government areas [13]. Similar patterns have also been observed in other countries, such as the Democratic Republic of the Congo and the Central African Republic [14]. If optimal quality SIA is not achieved, especially in areas with suboptimal surveillance performance, silent and undetectable transmissions are likely to continue, with possible spread of the variant to other geographical locations [15].

The quality of SIA is influenced by the effectiveness of the implementation process, which can be affected by multiple contextual factors that need to be better understood especially in terms of their dynamics [12,16,17,18]. In implementation science, it has since been recognised that contextual factors are highly potent in influencing the implementation success or failure of health interventions [19]. This has led to the evolution of determinants frameworks, which researchers and programme implementers can use to better understand the contextual factors [20]. Determinants framework provides a generalisable description of domains that influence implementation outcomes [20]. The advantage of using a determinants framework is that it enables the articulation of factors, whether facilitators or barriers, simply as independent variables [19]. Determinants frameworks, such as the Consolidated Framework for Implementation Research (CFIR), are commonly used tools to assess contextual issues associated with implementation success or failure [19,21].

Being a meta-theoretical framework developed through a synthesis of many theories, CFIR can account for a broad range of factors, making it a robust assessment tool [22]. CFIR categorises factors into five broad domains, and they include innovation, inner setting, outer setting, individual, and implementation process [19]. These domains are further subdivided into 47 constructs, which improve the degree to which diverse contextual factors are considered and defined [19]. Although CFIR can elucidate the multilevel nature of contextual factors, it may not provide explicit insights into how they link with each other within and across domains as expected in complex systems [19,20]. This can be achieved by leveraging systems thinking tools like causal loop diagrams to qualitatively illustrate the interconnectedness and interrelatedness of contextual factors [23].

Causal loop diagrams are useful for illustrating the dynamic interaction (interdependence and interconnectedness) that exist between contextual factors and can be used within the context of polio SIA [24]. Causal loop diagrams can allow qualitative mapping of the linkages between factors to generate an enhanced “whole-of-system” view [24,25]. The use of causal loop diagrams is rapidly growing as health systems stakeholders become increasingly aware of the impact of complexity on programmatic outcomes and the need for a broader systems perspective in addressing challenges [24,26].

Goal 2 of the *Polio Eradication Strategy (extended):2022–2029* is to stop cVDPV transmission and achieve the elimination of all types of variant polio circulation [2]. Moreover, in the face of a constricting financial landscape in global health, efforts to improve the efficiency of polio SIA are paramount. Therefore, this study aimed to explore and map the contextual factors that influence polio SIA implementation in the WHO African Region through a systems thinking approach.

## 2. Methods and Materials

### 2.1. Study Design

A rapid review method was used for this study based on the guidance provided by the Cochrane Rapid Review Methods Group [27]. Compared to other knowledge synthesis approaches like systematic review or scoping review, among others, a rapid review method can be conducted within a shorter period of time because its processes are more streamlined to produce quicker results for policy or practice decisions [27]. The main research question was the following: “What contextual factors influence polio supplementary immunisation activities in African countries and how do they interact with each other?”

### 2.2. Search Strategy

A comprehensive search was conducted to identify grey and published literature that described the factors that influence polio SIA in the African region. A search of two electronic databases, PubMed and Google Scholar, was conducted on 29th March 2024 without any date or language restrictions. A detailed search strategy was developed for PubMed and then adapted for Google Scholar. The search strategy consisted of keywords. These keywords were then combined using Boolean operators such as “OR”, “AND” and “NOT”. To improve the sensitivity of the keywords and ensure that the database search was as broad as possible, truncations were applied where necessary. Medical Subject Headings (MeSH) was specified for keywords when conducting the PubMed search so that all the references that have been indexed to them can be found. All Fields were specified for some keywords so that any literature where the term appeared can be identified. The final search string that was used is as follows: (“polio OR poliovirus OR poliomyelitis OR “Poliovirus vaccin*” OR “oral poliovirus vaccin*” OR “novel oral poliovirus vaccin* type 2” OR OPV OR nOPV2”) AND (“Supplement* OR “supplemental immunisation activit*” OR “supplemental immunisation activit*” OR SIA OR “immun* plus day*” OR IPD OR “national immun* day*” OR NID NOT (measles OR “vitamin A supplemen*”) AND (“lesson* OR experience* OR uptake OR use OR utiliz* OR access* OR accept* OR refus* OR willing* OR hesitancy OR program* OR strateg* OR factor* OR implement* OR determinant* OR introduc* OR bottleneck OR constraint* OR facilitat* OR barrier OR enable* OR drive*”). This string was geographically restricted to countries in the WHO African Region. In addition, the GPEI library was searched online [28], with a specific focus on the following reports:

(a) **Technical Advisory Group (TAG) Reports:** These are reports by independent experts tasked with reviewing progress towards polio eradication at the national and sub-national level.
(b) **Certification Reports:** These are reports by independent commissions at regional and global levels tasked with verifying polio eradication and overseeing the eradication certification process.
(c) **Annual reports of GPEI:** These are the annual reports of the GPEI.
(d) **Polio Transition Independent Monitoring Board (TIMB) Reports:** This board was created by GPEI to monitor and provide policy guidance related to polio transition. The board produces a report after each meeting.

### 2.3. Inclusion and Exclusion Criteria

The Sample, Phenomenon of Interest, Design, Evaluation, and Research type (SPIDER) framework was used to develop the criteria for inclusion. The inclusion criteria were as follows:

Sample: Countries in the WHO African Region;

Phenomenon of interest: Facilitators or barriers that influence the implementation of polio SIA;

Design: Case studies, observational, or experimental designs;

Evaluation: Programmatic reports (national or sub-national), community- or facility-based primary studies involving any stakeholders that play a role in polio SIA implementation;

Research type: Qualitative or quantitative studies or reports.

Studies were excluded if they were any of the following:

(a) Broad commentaries and viewpoints;
(b) Conducted outside of the WHO African Region.

### 2.4. Study Selection and Data Extraction

Duplicates were removed and two authors subsequently screened the titles and abstracts of 30% of the identified studies for relevance, while one author screened the remaining. A second author cross-checked the excluded studies for accuracy. Full texts of relevant studies were retrieved and reviewed by one author. A second author still verified the screened studies to ensure accuracy. Data were extracted from the included studies and reports using Microsoft Excel 365. A pre-tested data extraction tool was used. The details that were extracted from the studies included the following: author name, year of publication, country of study, study design, services integrated with SIA, delivery strategies, and factors. One author performed data extraction, while the second author cross-checked the final output for completeness. Relevant programme reports from the GPEI library were also checked. They included Technical Advisory Group reports for Horn of Africa (March 2010–November 2018), Nigeria (December 2005–October 2018), and Lake Chad (November 2017); Global Certification Commission meeting reports (February 1995–November 2023); GPEI annual reports (2005–2018); and Polio Transition Independent Monitoring Board reports (December 2010–September 2023).

### 2.5. Data Analysis

The total number of included literature articles were counted and categorised by year. The types of delivery strategies were summarised. Services that were integrated with SIAs in these countries were also summarised and classified into six categories. The extracted factors were analysed using a qualitative thematic analysis procedure [29]. The factors were read and re-read to develop themes. The descriptive themes generated were refined iteratively. During coding, efforts were made to preserve the original authors’ linguistic reasoning. Axial coding was performed to group the inductively generated themes into four categories: pre-SIA factors, peri-SIA factors, post-SIA factors, and crosscutting factors. Deductive reasoning was then used to map each factor to the domains and constructs of CFIR to reveal the multilevel nature of the contextual factors. The matrix of factors, based on CFIR domains and constructs, was presented in tables.

The linkages or connections between contextual factors influencing polio SIA were mapped using a causal loop diagram (CLD). In this diagram, arrows show the direction or influence between factors, with polarity indicated by (+) or (−). A positive (+) polarity meant that a change in one factor prompted a change in another in the same direction; a negative (−) polarity indicated a change in the opposite direction. Feedback loops were classified as either balancing (B) or reinforcing (R). A balancing loop signified that the variables’ changes offset each other, while a reinforcing loop signified that the changes amplified each other. Reinforcing loops could be either vicious, leading to negative outcomes, or virtuous, resulting in positive outcomes. Vensim PLE version 9.4.0 was used to develop the CLD [30].

## 3. Results

**Figure 1:**
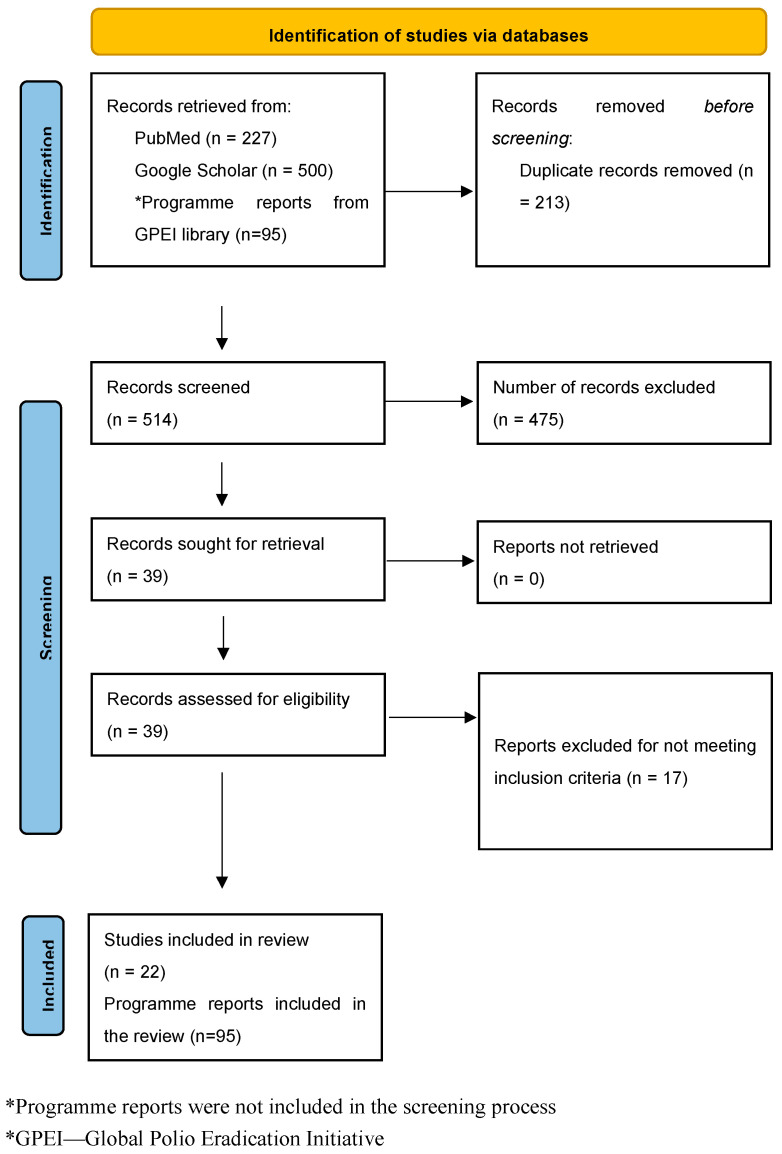
PRISMA flow diagram for the study.

The electronic database search yielded a total of 5587 outputs, with 227 from PubMed and 5360 from Google Scholar. However, only the first 500 records identified by Google Scholar, arranged in order of relevance, were considered [31]. After screening and assessment for eligibility, 22 peer-reviewed publications were included in the study. Additionally, 95 programme reports were considered: 31 from Technical Advisory Groups (TAG), 24 from the Global Certification Commission (GCC), 22 from the Polio Transition Independent Monitoring Board (TIMB), and 18 GPEI annual reports. Figure 1 shows the study’s flow chart.

### 3.1. Included Studies

The years of publication of the included studies range from 2002 to 2023. The studies covered nine countries in the African region, with eleven studies from Nigeria [13,17,32,33,34,35,36,37,38,39,40], three from South Sudan [12,41,42], two from Ethiopia [43,44], and one each from Ghana [45], Zambia [46], Cameroon [47], Tanzania [48], Guinea Bissau [16], and Kenya [18]. Additional details about the included studies are shown in Table 1.

### 3.2. Polio SIA Delivery Strategies and Service Integration

**Figure 2:**
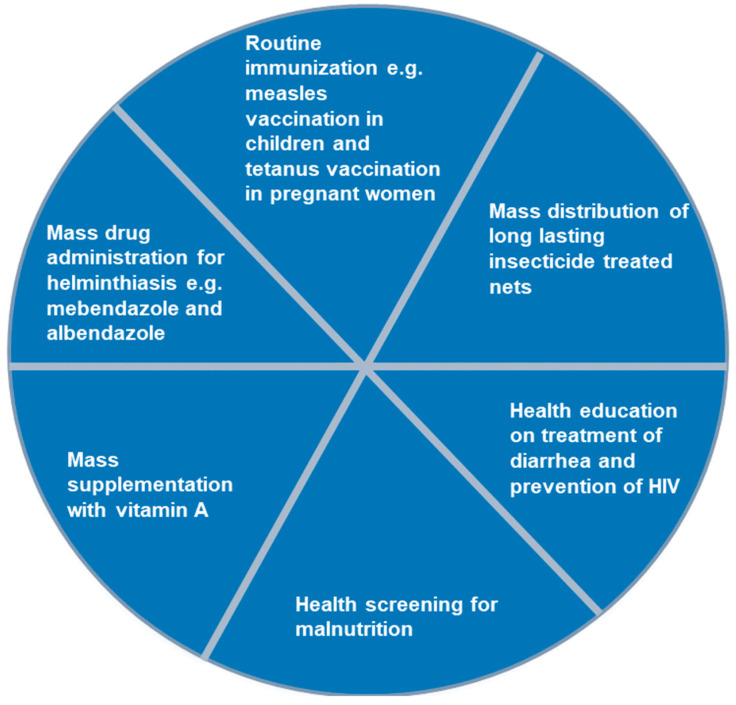
Services commonly integrated with polio supplementary immunisation activities in the Africa region, 2002–2023.

Some of the included studies reported polio SIA delivery strategies used within countries. The delivery strategies, mostly used in combination, included house-to-house [17,32,41,43,46,47], fixed post [18,32,47], school [17,47], hit and run [35,42], and special visits [18,32,43]. Other services are sometimes integrated with polio SIA in the countries, as reported in four studies [18,32,46,47]. As shown in Figure 2, services such as routine immunisation, mass drug administration for treatment of helminthiasis, mass supplementation with vitamin A, among others, were provided along with polio vaccination during SIA.

### 3.3. Contextual Factors Influencing Polio SIA in the WHO African Region

A total of 78 contextual factors that can influence polio SIA were identified across nine countries in the African region as shown on Table 2. These contextual factors were categorised into four as follows: pre-SIA, peri-SIA, post-SIA, and crosscutting. The pre-SIA factors are mainly related to vaccine availability, resource mobilisation, community buy-in, head of household support, health systems readiness, sociodemographic and economic characteristics of households, and trust. The peri-SIA factors are mainly related to compliance, access, incentivisation, and vaccination team performance. The post-SIA factors are related to evaluation and feedback. While the crosscutting factors are applicable at any time, they are related to community stewardship, political support, partner support, leadership, funding, and health systems’ resilience.

As shown in Table 3, the contextual factors identified match all the five domains of CFIR. The innovation domain had three (3.85%) factors, the outer setting domain had twenty (25.64%) factors, the inner setting domain had sixteen (20.51%) factors, the individual domain had twenty-six (33.33%) factors, and the implementation process domain had thirteen (16.67%) factors.

The factors in the innovation domain correspond to three constructs: the outer setting domain with five constructs, the inner setting domain with eight constructs, the individual domain with six constructs, and the implementation process domain with five constructs.

Innovation domain: The factors in this domain are related to the polio vaccine itself. Stakeholders’ decisions depend on WHO endorsement of the vaccine, which is based on the availability of strong evidence supporting its safety and effectiveness. The ease of oral administration was also highlighted.

Inner setting domain: The factors in this domain originate from where the SIA is conducted. They include the general characteristics of these settings such as terrain, infrastructure like cold chain capacity, communication, availability of a network of stakeholders to facilitate implementation, the extent of information sharing, and training, among others.

Outer setting domain: This domain identifies the factors that affect the functioning of the inner setting responsible for delivering the polio vaccination during SIA.

Individual domain: This domain considers the role and characteristics of the key actors like caregivers, heads of households, and health workers that influence uptake of the polio vaccine during SIA.

Implementation process domain: This domain covers the activities carried out to implement polio SIA.

### 3.4. Systems Mapping of the Contextual Factors Influencing Polio Supplementary Immunisation Activities in the WHO African Region

**Figure 3:**
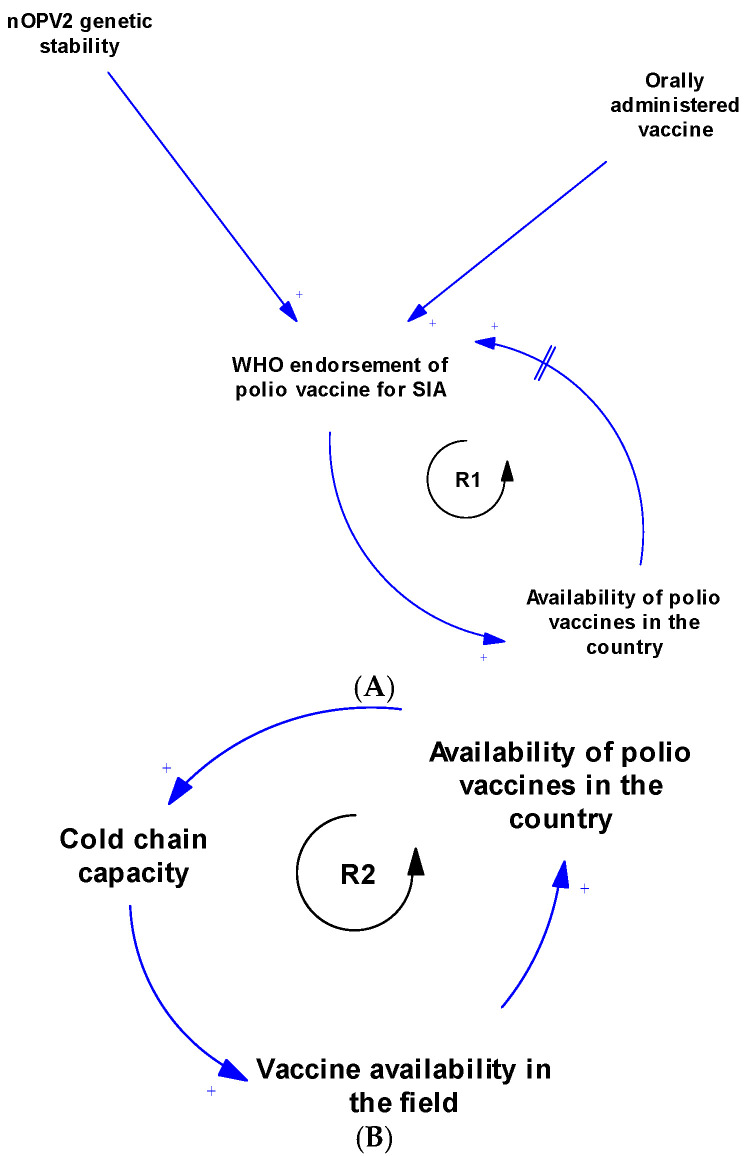

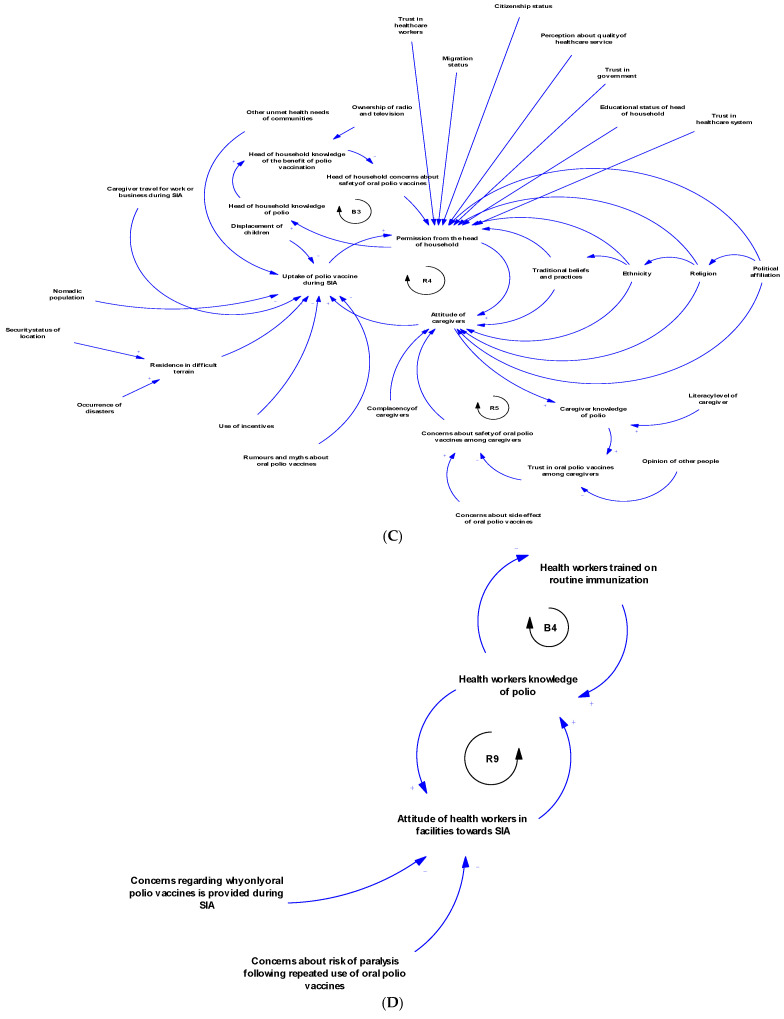
(**A**) Causal loop diagram of the relationship between WHO endorsement and availability of polio vaccines. (**B**) Causal loop diagram of the relationship between vaccine availability and cold chain capacity. (**C**) Causal loop diagram of factors responsible for compliance during SIA. (**D**) Causal loop diagram of the relationship between training, knowledge, and attitude of health workers towards polio supplementary immunisation activities.

**Figure 4:**
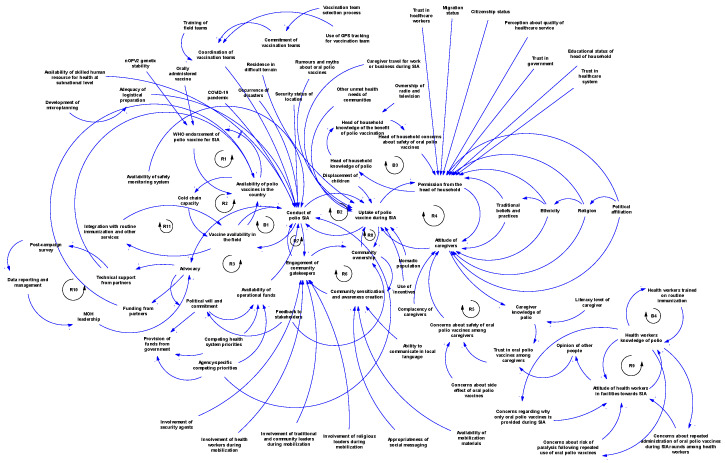
Full causal loop diagram of contextual factors influencing the implementation of polio supplementary immunisation activities in the WHO African Region.

There are multiple contingent and feedback connections between the contextual factors of polio SIA, as illustrated in Figure 3A–D and Figure 4. Figure 3A illustrates the feedback connection between the endorsement of a polio vaccine for SIA by the WHO and the availability of such vaccines within countries. Figure 3B shows that there is a feedback relationship between the availability of vaccine within country, the cold chain capacity, and the polio vaccine availability in the field. Figure 3C illustrates the feedback connection between permission from the head of household, the attitude of caregivers, and the uptake of polio vaccines during an SIA. However, permission from the head of the household is contingent upon several other factors, such as trust in health workers, health systems, and the government, as well as education level, migration status, and citizenship status. There is also a feedback connection between permission from the head of household and knowledge of polio. Additionally, caregiver attitudes depend on trust in the vaccine, which itself depends on opinions from others like health workers. Both permission from the head of household and caregiver attitudes towards polio vaccination are contingent upon sociodemographic factors like religion, ethnicity, political affiliation, and traditional beliefs and practices. Vaccine uptake is also influenced by rumours and myths about the polio vaccine, other unmet health needs, displacement of children, nomadic lifestyles, and use of incentives. Figure 3D shows the feedback connections between training of health workers on routine immunisation, knowledge of polio, and attitude towards SIA. As shown in Figure 4, there is a feedback loop between conducting SIA and the uptake of the polio vaccine during SIA. However, the conducting of SIA depends on many factors, including technical support from partners, available funding, human resources at the sub-national level, security conditions, and logistical planning, which itself depends on microplanning, among other factors. Also, there is another feedback connection that exists between the integration with routine immunisation and other services, and the availability of vaccines in the field. Other connections and feedback can be found in Figure 4 (the full causal loop diagram).

## 4. Discussion

This study aimed to explore and map the contextual factors influencing polio SIA implementation in the WHO African Region using a systems thinking approach and to offer insights into their dynamics. A total of 78 contextual factors were identified. These factors are time-dependent, with some being more prominent in the pre-SIA, peri-SIA, or post-SIA periods, while others are crosscutting and can affect polio SIA implementation at any time. The factors are distributed across the five domains of CFIR, indicating their multilevel nature. Furthermore, a qualitative systems map using a causal loop diagram demonstrated dynamic interactions between these contextual factors both within and across CFIR domains, generating feedback loops: eleven reinforcing loops and four balancing loops.

This study provided a comprehensive compilation of the contextual factors that can influence polio SIA in the African context. A closely related review that explored the socioecological challenges of SIA focused on Asia–Pacific countries [49]. Maintaining a deep knowledge of contextual factors is critical for polio programme stakeholders in Africa to inform efforts targeted at stopping transmission of cVDPVs, preventing future outbreaks of the variant, and maintaining the region’s certification status. The 22nd report of the Independent Monitoring Board of GPEI highlighted that, because of a complex mix of factors, the second goal of the Polio Eradication Strategy 2022–2029 (extended) is likely to be missed despite current efforts [50].

This study acknowledges the need for a systems thinking lens to broaden understanding of how the contextual factors that influence polio SIA link with each other. This integrated approach often results in systems-level outcomes [51]. The findings suggest that contextual factors are not static, but rather vary based on time of influence, level of influence, and dynamics of influence. Each of these elements (i.e., time, level, and dynamics) can play an important role in facilitating how strategies are designed and deployed to improve SIA implementation effectiveness.

The use of CFIR in this study enabled identified factors to be matched with theory-informed constructs to elucidate the level of influence [19]. We have shown that inner setting and individual domains had the most represented constructs. The prominence of individual-level factors related to caregivers in particular confirms the significance of household compliance in influencing the success or failure of polio SIA as also seen in other settings across the world [49,52]. CFIR is linked with the Expert Recommendations for Implementing Change (ERIC) [53], and thus polio programme implementers can make guided action-oriented decisions based on the identified factors of success or failure that they want to accentuate or resolve, respectively, in a particular setting. There are several examples of how CFIR has been used in this manner to strengthen health programmes [54,55]. In the United States, researchers collaboratively identified implementation barriers using CFIR and matched them with implementation strategies in the Expert Recommendations for Implementing Change (ERIC) tool to inform the development of a programme model to strengthen the implementation of primary healthcare for the older age group [54]. Examples of the use of CFIR in Africa across TB Programmes and in understanding and improving data quality have been reported [56,57].

This study mapped the connections between the identified contextual factors that influence polio SIA implementation using a causal loop diagram to provide insights into their dynamics of influence [58]. While CFIR enabled clear distinctions between multilevel contextual factors, applying a causal loop diagram allowed for the illustration of how these factors interconnect with one another. It was found that multiple linkages exist between factors within and across CFIR domains. Advancing a nuanced understanding of the dynamics of these factors is important for all stakeholders involved in polio programming in the African region, so that the policies and strategies they deploy to enhance implementation take a “whole-of-system” perspective.

The systems map generated showed that the contextual factors that influence polio SIA are interconnected and interdependent. The CLD revealed feedback loops that can serve as leverage points for interventions to improve the implementation effectiveness of SIA. Focusing on emerging loops ensures a tailored approach in implementation [59]. Loop R1 indicates that WHO endorsement of the polio vaccine is essential before vaccines can be used within a country. In turn, vaccine availability within countries further supports the endorsement process by generating data to aid decision-making. The delay mark highlights that this part of the loop takes time (and may consequently prolong circulation). Loop R2 illustrates the relationship between vaccine availability within the country, expansion of cold chain capacity, and subsequent vaccine availability in the field. Loop R3 depicts the advocacy system, emphasising the key role of advocacy in stimulating political will and commitment. With strong political commitment, the likelihood of fund releases increases, enabling polio SIA activities, which then reinforce advocacy efforts. The polio SIA compliance sub-system is represented by several loops, including R4, R5, and B3. This sub-system shows how various sociopolitical factors, trust in the health system and government, knowledge, and family dynamics influence vaccine uptake during SIA. Loops R6, R7, and R8 highlight the community stewardship loop, emphasising the critical role of community sensitisation and the involvement of community gatekeepers in operationalising SIA and vaccine uptake. Loops R9 and B4 constitute the key influencer sub-system, illustrating the dynamic relationship between health worker training on routine immunisation, knowledge of polio, and their attitudes towards SIA. Loop R10 shows the link between partner support in conducting post-campaign surveys and how data from these surveys inform further advocacy by the Ministry of Health leadership. Meanwhile, Loop R11 shows that if integration with routine immunisation and other services increases, polio vaccine availability in the field will increase, and, in turn, this will further strengthen integration efforts. These loops provide a clearer understanding of the complex interactions among multiple contextual factors that influence polio SIA. When designing strategies and innovations to improve SIA quality, decision-makers can precisely target specific loops while maintaining an overall perspective on how linked factors may respond.

### Implications for Policy and Practice

As the WHO African Region intensifies efforts towards polio eradication, the need for context-specific approaches to improve the implementation effectiveness of SIA has become even more essential. One way to support this is by increasing the capacity for using tools like causal loop diagrams among stakeholders, especially in the districts with persistently suboptimal polio SIA quality, to allow for a deeper exploration of factors. This can be achieved by strengthening the microplanning process to effectively employ a systems thinking perspective.

The polio SIA microplan has six main sections: resource estimate, cold chain logistics, operation, supervision, recording and reporting tools, and monitoring. Given the importance of contextual factors in influencing SIA implementation, an elaborate section on “context assessment” might be necessary. Expanding the microplan to include thorough context assessment will allow stakeholders to proactively consider the dynamics of the factors that are at play in their setting. Incorporating CLD in these context assessments can further empower stakeholders to have a better understanding of the linkages that exist between factors, including feedback loops, and, consequently, the leverage points for preemptive interventions during SIA preparation. This consolidates a paradigm shift in programming towards targeted, tailored, and localised solutioning. This important pre-implementation diagnostics and prioritisation exercise can support systems optimisation as it improves stakeholders’ ability to anticipate and manage emergence in their context. Several studies have reported instances where field implementers successfully used CLD for program re-design and prioritisation [60,61,62].

Another important consideration is that the inherent complexity of the contextual factors has potential implications for implementation fidelity [63]. A high degree of fit between SIA and the context in which they are conducted is essential. It may be difficult to ascertain whether field vaccination teams implement them, as intended, given the multitude of factors that act as moderators [63]. In the real world, field implementers can respond to exogenous complexities by “deliberately altering” an intervention design to accomplish their assigned tasks [64,65]. Therefore, tracking the fidelity of implementation of polio campaigns is important because it is linked with the expected outcome, which is the interruption of disease transmission in communities [63]. For polio SIA, implementation fidelity can be defined as “the degree to which a campaign is delivered, received and enacted as intended by the country polio programme” [63,66]. Fidelity data can enable programme and incident management teams to have a better understanding of the level of adherence of field vaccination teams to the polio SIA implementation protocol in specific settings.

There is a need to adapt post-campaign surveys, such as Lot Quality Assurance Sampling (LQAS), accordingly, to account for the inherent complexity of the contextual factors that influence polio SIA. However, LQAS rarely provides deep insights into how factors interact in that context to produce the observed outcome, whether success or failure. Therefore, a shift towards complexity-aware post-campaign assessments, including LQAS, is necessary so that findings are accompanied by a thorough description of the dynamics of factors in specific contexts. With this, programme and incident management teams can make better-informed decisions to address challenges when preparing for mop-up campaigns.

Another aspect of programme adaptation that should be prioritised, given the dynamics of identified factors, is service integration. This is a low-hanging opportunity to improve vaccination coverage for polio and other vaccine-preventable diseases (VPDs). The existing immunity gaps caused by persisting suboptimal essential vaccination coverage in the African Region, worsened by COVID-19 disruptions, apply to all VPDs and not polio alone. This layering of immunity gaps creates a favourable condition for multiple and simultaneous outbreaks [67]. Thus, institutionalising integrated campaigns is a programmatic imperative for all countries in the region. In this post-COVID-19 period, coupled with present funding shortages, it is no longer rational to deploy resources for mono-antigen SIA. Polio SIA can also be used as an opportunity to deliver other key child survival interventions, such as long-lasting insecticide treated nets and de-worming tablets, among others, in the households that are visited. Furthermore, there is an intersection between the at-risk cohort of targeted children for vaccination during SIA and mass public health campaigns for the distribution of drugs aimed at controlling malaria and neglected tropical diseases. Therefore, these campaigns can be utilised to address multiple health needs simultaneously. Examples of some integration efforts within the polio programme have been documented in Africa, including how they are perceived by stakeholders [68,69,70,71]. Countries can consider actively exploring and utilising all these opportunities for providing vaccination and vice versa. Adopting an integration policy (for service delivery) advances synergies between health programmes, but importantly, improves the efficiency of resource utilisation. Indeed, there are operational issues to contend with that can set back progress on programme alignment; however, there are already country experiences with integration that others can learn from [18,32,46,47]. Besides, this should be a gradual process to allow adequate programme learning and course-correction adapted to context.

The findings from this study should be interpreted with some limitations in mind. Only 22 studies from across nine countries were included in this study. As such, the result should be generalised with caution. Nonetheless, programme reports were also considered. Since this rapid review focused specifically on identifying contextual factors and highlighting evidence gaps, an appraisal of the quality of the included publications was not deemed relevant. Also, only the first 500 results from Google Scholar were selected. Although unlikely, it might be possible that some publications were missed. The screening and data extraction process was streamlined and mostly conducted by one author. However, the contextual factors that were identified from these publications were comprehensive. The causal loop diagram was developed using secondary data extracted from published studies; as such, some factors and linkages might not be represented. For this reason, more primary studies are recommended. The CLD was constructed by one author and validated by others; as such, the building process can be prone to unconscious bias. However, the authors are experienced in immunisation programmes and polio programming. It is essential to note that the CLD is not intended to be generalisable across diverse settings. Programme implementers are encouraged to adjust their own qualitative maps of contextual factors to reflect the dynamics in their context at that point in time.

## 5. Conclusions

Although most countries in the African region have interrupted the transmission of cVDPVs, a few sub-national settings in a limited number of countries continue to struggle with suboptimal immunisation response activities, resulting in ongoing circulation of variant polio serotypes. Unfortunately, this poses a serious risk to other settings within the same country and even other countries; as such, efforts to enhance SIA quality are an urgent priority. This review identified the multilevel nature of the contextual factors that influence polio SIA using CFIR and described the dynamics of these factors using CLD. The integration of CLD and CFIR in this study offers critical insights into the potential feedback loops that exist between the contextual factors, which can be used as leverage points for policy and practice improvements. Given the complexity of the contextual factors that influence polio SIA, a “one-size-fits-all” policy for diverse contexts might not be beneficial. Instead, strategies need to be tailored to specific contexts depending on the prevailing factors that are at play at that point in time.

### Implications for Future Research

There is a need for more evidence on the contextual factors that influence polio SIA from specific settings across African countries. Evidence can be generated from existing data within countries or primary studies that focus specifically on sub-national settings that persistently record suboptimal SIA quality in the WHO African Region. Primary studies that employ rapid ethnographic assessments can help to document the SIA implementation process, observe implementation fidelity, and investigate contextual factors that might have been missed in this review especially in regions with ongoing active transmission of variant polio viruses. This should inform implementation studies to assess conceptually backed strategies for improving the implementation success of polio SIA in the African region.

## Figures and Tables

**Table 1:** Studies included in the review.

Author	Year of Publication	Country	Study Design
Browne et al. [45]	2002	Ghana	Cross-sectional design
Arulogun et al. [38]	2007	Nigeria	Cross-sectional design
Ghinai et al. [34]	2013	Nigeria	Cross-sectional design
Michael et al. [39]	2014	Nigeria	Cross-sectional design
Mohammed et al. [40]	2014	Nigeria	Cross-sectional design
Babaniyi et al. [46]	2014	Zambia	Cross-sectional design
Gammino et al. [17]	2014	Nigeria	Cross-sectional design
Onyeka et al. [32]	2014	Nigeria	Cross-sectional design
Haskew [41]	2015	South Sudan	Cross-sectional design
Haddison et al. [47]	2018	Cameroon	Cross-sectional design
Umeh [33]	2018	Nigeria	Cross-sectional design
Nkwogu et al. [35]	2018	Nigeria	Cross-sectional design
Iyal et al. [36]	2018	Nigeria	Cross-sectional design
Tegegne et al. [44]	2018	Ethiopia	Cross-sectional design
Mohamed et al. [48]	2020	Tanzania	Cross-sectional design
Buus et al. [16]	2021	Guinea-Bissau	Cross-sectional design
Aliyu et al. [37]	2021	Nigeria	Cross-sectional design
Maleghemi et al. [42]	2022	South Sudan	Cross-sectional design
Harvey et al. [18]	2022	Kenya	Cross-sectional design
Kidanne et al. [43]	2022	Ethiopia	Cross-sectional design
Tegegne et al. [12]	2023	South Sudan	Cross-sectional design
Asekun et al. [13]	2023	Nigeria	Cross-sectional design

**Table 2:** Contextual factors influencing polio supplementary immunisation activities in the WHO African region.

Pre-SIA Factors	Peri-SIA Factors	Post-SIA Factors	Crosscutting Factors
WHO endorsement of vaccine	Use of incentives	Feedback to stakeholders	Community sensitisation and awareness creation
Orally administered vaccine	Use of GPS tracking for vaccination team	Data reporting and management	Community ownership
nOPV2 genetic stability	Vaccine availability in the field	Post-campaign survey	Ability to communicate in local language
Permission from the head of household	Availability of safety monitoring system		COVID-19 pandemic
Trust in healthcare system	Displacement of children		Technical support from partners
Availability of mobilisation materials	Rumours and myths about oral polio vaccines		Funding from partners
Development of microplanning	Trust in oral polio vaccines among caregivers		Occurrence of disasters
Availability of operation funds	Commitment of vaccination teams		MOH leadership
Training of field teams	Coordination of vaccination teams		Political will and commitment
Engagement of community gatekeepers	Concerns about safety of oral polio vaccines among caregivers		Security status of location
Availability of skilled human resources for health at sub-national level	Concerns about repeated administration of oral polio vaccines during SIA rounds among health workers		
Provision of funds from government	Concerns regarding why only oral polio vaccines are provided during SIA		
Availability of polio vaccines in the country	Concerns about side effects of oral polio vaccines		
Competing health system priorities	Concerns about risk of paralysis following repeated use of oral polio vaccines		
Agency-specific competing priorities	Integration of routine immunisation and other services		
Cold chain capacity	Opinion of close relatives		
Trust in healthcare workers	Other unmet health needs of communities		
Trust in government	Nomadic population		
Advocacy	Residence in difficult terrain		
Attitude of caregivers	Caregiver travel for work or business during SIA		
Ethnicity			
Religion			
Political affiliation			
Vaccination team selection process			
Appropriateness of social messaging			
Literacy level of caregiver			
Traditional beliefs and practices			
Migration			
Citizenship of the country			
Perception about quality of healthcare service			
Involvement of security agents			
Caregiver knowledge of poliomyelitis			
Adequacy of logistical preparation			
Involvement of religious leaders during mobilisation			
Involvement of health workers during mobilisation			
Involvement of traditional and community leaders during mobilisation			
Ownership of radio and television			
Complacency of caregivers			
Educational status of head of household			
Head of household knowledge of the benefit of polio vaccination			
Head of household knowledge of polio			
Head of household concerns about safety of oral polio vaccines			
Health workers trained on routine immunisation			
Attitude of health workers in facilities towards SIA			
Health workers’ knowledge of polio			

WHO—World Health Organization. nOPV2—Novel oral polio vaccine type 2. SIA—Supplementary Immunisation Activities. MOH—Ministry of Health.

**Table 3:** CFIR constructs of the contextual factors influencing polio supplementary immunisation activities in the WHO African region.

CFIR Domains	CFIR Constructs	Identified Factors
**Innovation**		
	Innovation evidence base	WHO endorsement of vaccine
	Innovation complexity	Orally administered vaccine
	Innovation relative advantage	nOPV2 genetic stability
**Outer setting**		
	Local condition	Political will and commitment
	Financing	Provision of funds from government
	Policies and laws	Vaccine availability in the country
	Critical incident	Competing health system priorities
	Critical incident	Agency-specific competing priorities
	Local condition	Ethnicity
	Local condition	Religion
	Local condition	Political affiliation
	Critical incident	Security status of location
	Local attitude	Traditional beliefs and practices
	Local condition	Migration
	Local condition	Citizenship of the country
	Local condition	Displacement of children
	Local attitude	Rumours and myths about oral polio vaccine
	Local condition	Nomadic population
	Critical incident	COVID-19 pandemic
	Partnerships and connections	Technical support from partners
	Financing	Funding from partners
	Critical incident	Occurrence of disasters
	Local condition	MOH leadership
**Inner setting**		
	Available resources	Availability of operation funds
	Available resources	Availability of skilled human resources for health at sub-national level
	Information technology infrastructure	Use of GPS tracking for vaccination team
	Access to knowledge and information	Health workers trained on routine immunisation
	Access to knowledge and information	Training of field teams
	Work infrastructure	Cold chain capacity
	Relational connection	Involvement of religious leaders during mobilisation
	Relational connection	Involvement of health workers during mobilisation
	Relational connection	Involvement of traditional and community leaders during mobilisation
	Incentives	Use of incentives
	Available resources	Vaccine availability in the field
	Structural characteristics	Residence in difficult terrain
	Culture	Community ownership
	Available resources	Availability of mobilisation materials
	Communication	Appropriateness of social messaging
	Communication	Ability to communicate in local language
**Individuals**		
	Innovation recipients	Permission from the head of household
	Innovation recipients	Trust in healthcare system
	Innovation recipients	Trust in healthcare workers
	Innovation recipients	Trust in government
	Innovation recipients	Attitude of caregivers
	Innovation recipients	Literacy level of caregiver
	Innovation recipients	Perception about quality of healthcare service
	Innovation recipients	Caregiver knowledge of poliomyelitis
	Innovation recipients	Ownership of radio and television
	Innovation recipients	Complacency of caregivers
	Innovation recipients	Educational status of head of household
	Innovation recipients	Head of household knowledge of the benefit of polio vaccination
	Innovation recipients	Head of household knowledge of polio
	Innovation recipients	Head of household concerns about safety
	Innovation recipients	Trust in oral polio vaccine among caregivers
	Innovation recipients	Concerns about safety of oral polio vaccines among caregivers
	Innovation recipients	Concerns regarding why only oral polio vaccines are provided during SIA
	Innovation recipients	Concerns about side effects of oral polio vaccines
	Innovation recipients	Concerns about risk of paralysis following repeated use of oral polio vaccines
	Implementation facilitators	Attitude of health workers in facilities towards SIA
	Implementation facilitators	Health workers’ knowledge of polio
	Implementation facilitators	Concerns about repeated administration of oral polio vaccines during SIA rounds among health workers
	Opinion leaders	Opinion of close relatives
	Opportunity	Caregiver travel for work or business during SIA
	Motivation	Commitment of vaccination teams
	Need	Other unmet health needs of communities
**Implementation process**		
	Planning	Development of microplanning
	Teaming	Engagement of community gatekeepers
	Engaging	Advocacy
	Engaging	Vaccination team selection process
	Tailoring strategies	Involvement of security agents
	Teaming	Coordination of vaccination teams
	Reflecting and evaluating	Feedback to stakeholders
	Reflecting and evaluating	Data reporting and management
	Reflecting and evaluating	Post-campaign survey
	Tailor strategies	Community sensitisation and awareness creation
	Tailor strategies	Integration of routine immunisation and other services
	Planning	Adequacy of logistical preparation
	Planning	Availability of safety monitoring system

## Data Availability

Data are contained within the article.

## References

[1] Tediosi F., Villa S., Levison D., Ekeman E., Politi C. (2024). Leveraging Global Investments for Polio Eradication to Strengthen Health Systems’ Resilience through Transition. Health Policy Plan..

[2] World Health Organization (2021). Polio Eradication Strategy 2022–2026: Delivering on a Promise.

[3] Cochi S.L., Pallansch M.A. (2021). The Long and Winding Road to Eradicate Vaccine-Related Polioviruses. J. Infect. Dis..

[4] Mohanty A., Rohilla R., Zaman K., Hada V., Dhakal S., Shah A., Padhi B.K., Al-Qaim Z.H., Altawfiq K.J.A., Tirupathi R. (2023). Vaccine Derived Poliovirus (VDPV). Le Infez. Med..

[5] World Health Organization Statement of the Twenty-Seventh Polio IHR Emergency Committee. https://www.who.int/news/item/19-02-2021-statement-of-the-twenty-seventh-polio-ihr-emergency-committee.

[6] World Health Organization (2022). Standard Operating Procedures: Responding to a Poliovirus Event or Outbreak, Version 4.1..

[7] Global Polio Eradication Initiative (2024). GPEI Press Release on nOPV2 Prequalification.

[8] Ochoge M., Futa A.C., Umesi A., Affleck L., Kotei L., Daffeh B., Saidy-Jah E., Njie A., Oyadiran O., Edem B. (2024). Safety of the Novel Oral Poliovirus Vaccine Type 2 (nOPV2) in Infants and Young Children Aged 1 to <5 Years and Lot-to-Lot Consistency of the Immune Response to nOPV2 in Infants in The Gambia: A Phase 3, Double-Blind, Randomised Controlled Trial. Lancet.

[9] Te Yeh M., Bujaki E., Dolan P.T., Smith M., Wahid R., Konz J., Weiner A.J., Bandyopadhyay A.S., Van Damme P., De Coster I. (2020). Engineering the Live-Attenuated Polio Vaccine to Prevent Reversion to Virulence. Cell Host Microbe.

[10] Brown A.E., Okayasu H., Nzioki M.M., Wadood M.Z., Chabot-Couture G., Quddus A., Walker G., Sutter R.W. (2014). Lot Quality Assurance Sampling to Monitor Supplemental Immunization Activity Quality: An Essential Tool for Improving Performance in Polio Endemic Countries. J. Infect. Dis..

[11] World Health Organization (2018). Best Practice for Monitoring the Quality of Polio Eradication Campaign Performance.

[12] Tegegne A.A., Anyuon A.N., Legge G.A., Ferede M.A., Isaac Z., Laku K.A., Biadgilign S., Kilo O.T.D., Ndenzako F., Modjirom N. (2023). A Circulating Vaccine-Derived Poliovirus Type 2 Outbreak in a Chronic Conflict Setting: A Descriptive Epidemiological Study in South Sudan—2020 to 2021. BMC Infect. Dis..

[13] Asekun A., Nkwogu L., Bawa S., Usman S., Edukugho A., Ocheh J., Banda R., Nganda G.W., Nsubuga P., Archer R. (2023). Deployment of Novel Oral Polio Vaccine Type 2 under Emergency Use Listing in Nigeria: The Rollout Experience. Pan Afr. Med. J..

[14] Alleman M.M., Jorba J., Riziki Y., Henderson E., Mwehu A., Seakamela L., Howard W., Kadiobo Mbule A., Nsamba R.N., Djawe K. (2023). Vaccine-Derived Poliovirus Serotype 2 Outbreaks and Response in the Democratic Republic of the Congo, 2017–2021. Vaccine.

[15] Auzenbergs M., Fountain H., Macklin G., Lyons H., O’Reilly K.M. (2023). The Impact of Surveillance and Other Factors on Detection of Emergent and Circulating Vaccine Derived Polioviruses. Gates Open Res..

[16] Buus M., da Silva I., Nielsen S., Thysen S.M., Fisker A.B. (2021). Coverage and Factors Associated with Receiving Campaign Polio Vaccines in an Urban Population in Guinea-Bissau. Vaccine.

[17] Gammino V.M., Nuhu A., Gerber S., Gasasira A., Sugerman D.E., Manneh F., Chenoweth P., Kurnit M.R., Abanida E.A. (2014). An Evaluation of Polio Supplemental Immunization Activities in Kano, Katsina, and Zamfara States, Nigeria: Lessons in Progress. J. Infect. Dis..

[18] Harvey B., Dalal W., Amin F., McIntyre E., Ward S., Merrill R.D., Mohamed A., Hsu C.H. (2022). Planning and Implementing a Targeted Polio Vaccination Campaign for Somali Mobile Populations in Northeastern Kenya Based on Migration and Settlement Patterns. Ethn. Health.

[19] Damschroder L.J., Reardon C.M., Widerquist M.A.O., Lowery J. (2022). The Updated Consolidated Framework for Implementation Research Based on User Feedback. Implement. Sci..

[20] Nilsen P. (2020). Making Sense of Implementation Theories, Models, and Frameworks. Implementation Science 3.0.

[21] Skolarus T.A., Lehmann T., Tabak R.G., Harris J., Lecy J., Sales A.E. (2017). Assessing Citation Networks for Dissemination and Implementation Research Frameworks. Implement. Sci..

[22] Damschroder L.J., Aron D.C., Keith R.E., Kirsh S.R., Alexander J.A., Lowery J.C. (2009). Fostering Implementation of Health Services Research Findings into Practice: A Consolidated Framework for Advancing Implementation Science. Implement. Sci..

[23] Roxas F.M.Y., Rivera J.P.R., Gutierrez E.L.M. (2019). Locating Potential Leverage Points in a Systems Thinking Causal Loop Diagram toward Policy Intervention. World Futures.

[24] Adam T., de Savigny D. (2012). Systems Thinking for Strengthening Health Systems in LMICs: Need for a Paradigm Shift. Health Policy Plan..

[25] Gomersall T. (2018). Complex Adaptive Systems: A New Approach for Understanding Health Practices. Health Psychol. Rev..

[26] Baugh Littlejohns L., Hill C., Neudorf C. (2021). Diverse Approaches to Creating and Using Causal Loop Diagrams in Public Health Research: Recommendations From a Scoping Review. Public Health Rev..

[27] Garritty C., Gartlehner G., Nussbaumer-Streit B., King V.J., Hamel C., Kamel C., Affengruber L., Stevens A. (2021). Cochrane Rapid Reviews Methods Group Offers Evidence-Informed Guidance to Conduct Rapid Reviews. J. Clin. Epidemiol..

[28] World Health Organization Library—GPEI. https://polioeradication.org/library/.

[29] Clarke V., Braun V., Hayfield N. (2015). Thematic Analysis. Qualitative Psychology: A Practical Guide to Research Methods.

[30] Sapiri H., Zulkepli J., Ahmad N., Abidin N.Z., Hawari N.N. (2017). Introduction to System Dynamic Modelling and Vensim Software: UUM Press.

[31] Haddaway N.R., Collins A.M., Coughlin D., Kirk S. (2015). The Role of Google Scholar in Evidence Reviews and Its Applicability to Grey Literature Searching. PLoS ONE.

[32] Onyeka I.N., Ilika A.L., Ilika F.N., Umeh D.C., Onyibe R.I., Okoye C.J., Diden G., Onubogu C.U. (2014). Experiences from Polio Supplementary Immunization Activities in Anambra State, Nigeria. Niger. J. Clin. Pract..

[33] Umeh G.C., Nomhwange T.I., Shamang A.F., Zakari F., Musa A.I., Dogo P.M., Gugong V., Iliyasu N. (2018). Attitude and Subjective Wellbeing of Non-Compliant Mothers to Childhood Oral Polio Vaccine Supplemental Immunization in Northern Nigeria. BMC Public Health.

[34] Ghinai I., Willott C., Dadari I., Larson H.J. (2013). Listening to the Rumours: What the Northern Nigeria Polio Vaccine Boycott Can Tell Us Ten Years On. Glob. Public Health.

[35] Nkwogu L., Shuaib F., Braka F., Mkanda P., Banda R., Korir C., Bawa S., Mele S., Saidu M., Mshelia H. (2018). Impact of Engaging Security Personnel on Access and Polio Immunization Outcomes in Security-Inaccessible Areas in Borno State, Nigeria. BMC Public Health.

[36] Iyal H.A., Shuaib F., Dauda M., Suleiman A., Braka F., Tegegne S.G., Nsubuga P., Nomhwange T., Yehualashet Y.G., Ishaku S. (2018). Assessment of Unmet Needs to Address Noncompliant Households during Polio Supplemental Immunization Activities in Kaduna State, 2014–2016. BMC Public Health.

[37] Aliyu N., Bawa M.K., Gidado S., Ohuabunwo C., Esapa L., Archer W.R., Sule A., Bolatito H.A., Mamman A., Olayinka A. (2021). Revelation of an Important Weakness in Polio Elimination Efforts in Nigeria: A Descriptive Cross-Sectional Study of Nomadic Dynamics in Sokoto and Taraba States, May 2013. Pan Afr. Med. J..

[38] Arulogun O.S., Obute J.A. (2007). Health Workers’ Perception about the Supplemental Immunization Activities in Gombe Local Government Area, Gombe State. Afr. J. Med. Med. Sci..

[39] Michael C.A., Ashenafi S., Ogbuanu I.U., Ohuabunwo C., Sule A., Corkum M., Mackay S., Storms A.D., Achari P., Biya O. (2014). An Evaluation of Community Perspectives and Contributing Factors to Missed Children During an Oral Polio Vaccination Campaign—Katsina State, Nigeria. J. Infect. Dis..

[40] Mohammed A., Sabitu K., Nguku P., Abanida E., Sheidu S., Dalhat M., Dankoli R., Gidado S., Suleiman I. (2014). Characteristics of Persons Refusing Oral Polio Vaccine during the Immunization plus Days—Sokoto, Nigeria 2011. Pan Afr. Med. J..

[41] Haskew J., Kenyi V., William J., Alum R., Puri A., Mostafa Y., Davis R. (2015). Use of Mobile Information Technology during Planning, Implementation and Evaluation of a Polio Campaign in South Sudan. PLoS ONE.

[42] Maleghemi S., Tegegne A.A., Ferede M., Bassey B.E., Akpan G.U., Bello I.M., Ticha J.M., Anyuon A., Waya J.L., Okiror S.O. (2022). Polio Eradication in a Chronic Conflict Setting Lessons from the Republic of South Sudan, 2010–2020. Pan Afr. Med. J..

[43] Kidanne L., Bisrat F., Mohammed M., Deyessa N. (2022). Curbing an Outbreak of Circulating Vaccine Derived Poliovirus Type 2 in Dollo Zone, Somali Region, Ethiopia: Response to Outbreak. Pan Afr. Med. J..

[44] Tegegne A.A., Braka F., Shebeshi M.E., Aregay A.K., Beyene B., Mersha A.M., Ademe M., Muhyadin A., Jima D., Wyessa A.B. (2018). Characteristics of Wild Polio Virus Outbreak Investigation and Response in Ethiopia in 2013–2014: Implications for Prevention of Outbreaks Due to Importations. BMC Infect. Dis..

[45] Browne E.N.L., Bonney A.A., Agyapong F.A., Essegbey I.T. (2002). Factors Influencing Participation in National Immunization Days in Kumasi, Ghana. Ann. Trop. Med. Parasitol..

[46] Babaniyi O., Siziya S., Mukonka V., Kalesha P., Mutambo H., Matapo B., Musanje H. (2013). Child Nutrition and Health Campaign in 2012 in Zambia: Coverage Rates for Measles, Oral Polio Vaccine, Vitamin A, and De-Worming. Open Vaccine J..

[47] Haddison E.C., Ngono D., Kouamen G.T., Kagina B.M. (2018). Successful Polio Supplementary Immunisation Activities in a Security Compromised Zone—Experiences from the Southwest Region of Cameroon. Vaccine.

[48] Mohamed N., Simba D., Mphuru A., Lyimo D., Kyesi F. (2020). Lessons Learned in the Implementation of Supplementary Immunization Activity (SIA) Field Guidelines for Injectable Vaccines—Experiences from Tanzania. Vaccine.

[49] Ahmad H., Sanef S.A., Shahabudin W.Z., Mohtar N., Hassan M.R., Jeffree M.S., Lukman K.A., Ghazi H.F., Syed Abdul Rahim S.S. (2023). Socioecological Challenges of Polio Supplementary Immunization Activities (SIAs) in the Asia-Pacific Region: A Systematic Review. J. Environ. Public Health.

[50] Independent Monitoring Board of the Global Polio Eradication Initiative. 22nd Report of the Independent Monitoring Board of Global Polio Eradication Initiative. https://polioeradication.org/wp-content/uploads/2023/09/22nd-Report-of-The-Independent-Monitoring-Board-IMB.pdf.

[51] Whelan J., Fraser P., Bolton K.A., Love P., Strugnell C., Boelsen-Robinson T., Blake M.R., Martin E., Allender S., Bell C. (2023). Combining Systems Thinking Approaches and Implementation Science Constructs within Community-Based Prevention: A Systematic Review. Health Res. Policy Syst..

[52] Molodecky N.A., Usman A., Javaid A., Wahdan A., Parker E.P., Ahmed J.A., Shah N., Agbor J., Mahamud A., Safdar R.M. (2021). Quantifying Movement Patterns and Vaccination Status of High Risk Mobile Populations in Pakistan and Afghanistan to Inform Poliovirus Risk and Vaccination Strategy. Vaccine.

[53] Powell B.J., Waltz T.J., Chinman M.J., Damschroder L.J., Smith J.L., Matthieu M.M., Proctor E.K., Kirchner J.E. (2015). A Refined Compilation of Implementation Strategies: Results from the Expert Recommendations for Implementing Change (ERIC) Project. Implement. Sci..

[54] Shin M.H., Montano A.-R.L., Adjognon O.L., Harvey K.L.L., Solimeo S.L., Sullivan J.L. (2023). Identification of Implementation Strategies Using the CFIR-ERIC Matching Tool to Mitigate Barriers in a Primary Care Model for Older Veterans. Gerontol..

[55] Weir A., Presseau J., Kitto S., Colman I., Hatcher S. (2021). Strategies for Facilitating the Delivery of Cluster Randomized Trials in Hospitals: A Study Informed by the CFIR-ERIC Matching Tool. Clin. Trials.

[56] Donessouné F.M.G., Sossa O.G., Kouanda S. (2024). Using CFIR Framework for Understanding Barriers and Facilitators to Implementation of Community Tuberculosis Program in Burkina Faso. Front. Health Serv..

[57] Gimbel S., Mwanza M., Nisingizwe M.P., Michel C., Hirschhorn L., the AHI PHIT Partnership Collaborative (2017). Improving Data Quality across 3 Sub-Saharan African Countries Using the Consolidated Framework for Implementation Research (CFIR): Results from the African Health Initiative. BMC Health Serv. Res..

[58] Baugh Littlejohns L., Baum F., Lawless A., Freeman T. (2018). The Value of a Causal Loop Diagram in Exploring the Complex Interplay of Factors That Influence Health Promotion in a Multisectoral Health System in Australia. Health Res. Policy Syst..

[59] Penney L.S., Damush T.M., Rattray N.A., Miech E.J., Baird S.A., Homoya B.J., Myers L.J., Bravata D.M. (2021). Multi-Tiered External Facilitation: The Role of Feedback Loops and Tailored Interventions in Supporting Change in a Stepped-Wedge Implementation Trial. Implement. Sci. Commun..

[60] Kang H., Nembhard H.B., Curry W., Ghahramani N., Hwang W. (2017). A Systems Thinking Approach to Prospective Planning of Interventions for Chronic Kidney Disease Care. Health Syst..

[61] Schoenenberger L.K., Bayer S., Ansah J.P., Matchar D.B., Mohanavalli R.L., Lam S.S., Ong M.E. (2016). Emergency Department Crowding in Singapore: Insights from a Systems Thinking Approach. SAGE Open Med..

[62] Rehbock C., Krafft T., Sommer A., Beumer C., Beckers S.K., Thate S., Kaminski J., Ziemann A. (2023). Systems Thinking Methods: A Worked Example of Supporting Emergency Medical Services Decision-Makers to Prioritize and Contextually Analyse Potential Interventions and Their Implementation. Health Res. Policy Syst..

[63] Carroll C., Patterson M., Wood S., Booth A., Rick J., Balain S. (2007). A Conceptual Framework for Implementation Fidelity. Implement. Sci..

[64] Moore J.E., Bumbarger B.K., Cooper B.R. (2013). Examining Adaptations of Evidence-Based Programs in Natural Contexts. J. Prim. Prev..

[65] Lara M., Bryant-Stephens T., Damitz M., Findley S., González Gavillán J., Mitchell H., Ohadike Y.U., Persky V.W., Ramos Valencia G., Rojas Smith L. (2011). Balancing “Fidelity” and Community Context in the Adaptation of Asthma Evidence-Based Interventions in the “Real World”. Health Promot. Pract..

[66] Ahmad S.Z., Ivers N., Zenlea I., Parsons J.A., Shah B.R., Mukerji G., Punthakee Z., Shulman R. (2024). An Assessment of Adaptation and Fidelity in the Implementation of an Audit and Feedback-Based Intervention to Improve Transition to Adult Type 1 Diabetes Care in Ontario, Canada. Implement. Sci. Commun..

[67] Omosigho P.O., Okesanya O.J., Olaleke N.O., Eshun G., Lucero-Prisno III D.E. (2023). Multiple Burden of Infectious Disease Outbreaks: Implications for Africa Healthcare System. J. Taibah Univ. Med. Sci..

[68] Craig A.S., Haydarov R., O’Malley H., Galway M., Dao H., Ngongo N., Baranyikwa M.T., Naqvi S., Abid N.S., Pandak C. (2017). The Public Health Legacy of Polio Eradication in Africa. J. Infect. Dis..

[69] Chehab E.T., Anya B.-P.M., Onyango A.W., Tevi-Benissan M.C., Okeibunor J., Mkanda P., Mihigo R. (2016). Experience of Integrating Vitamin A Supplementation into Polio Campaigns in the African Region. Vaccine.

[70] Closser S., Neel A.H., Gerber S., Alonge O. (2024). From Legacy to Integration in the Global Polio Eradication Initiative: Looking Back to Look Forward. BMJ Glob. Health.

[71] Ahmed S.T., Haider S.S., Hanif S., Anwar H.B., Mehjabeen S., Closser S., Bazant E., Sarker M. (2023). A Scoping Review on Integrated Health Campaigns for Immunization in Low-and Middle-Income Countries. Health Policy Plan..

